# Factors associated with attention-deficit/hyperactivity disorder among Tunisian children

**DOI:** 10.3389/fpsyt.2025.1462099

**Published:** 2025-02-07

**Authors:** Asma Guedria, Mohamed Guedria, Manel Ben Fredj, Randaline Ayoub, Hela Ben Abid, Ahmed Mhalla, Hela Slama

**Affiliations:** ^1^ Faculty of Medicine, University of Monastir, Monastir, Tunisia; ^2^ Department of Child and Adolescent Psychiatry, Fattouma Bourguiba University Hospital, Monastir, Tunisia; ^3^ Research Laboratory “Vulnerability to Psychotic Disorders – LR05ES10”, Faculty of Medicine, University of Monastir, Monastir, Tunisia; ^4^ Department of Preventive Medicine and Infection Control, Hospital Haj Ali Soua of Ksar-Hellal, Monastir, Tunisia; ^5^ Depatement of Psychiatry, Fattouma Bourguiba University Hospital, Monastir, Tunisia; ^6^ Departmental Hospital Center La Candélie, Child and Adolescent Psychiatry, Pont-du-Casse, France

**Keywords:** ADHD, psychosocial, environmental, predisposition, risk factors

## Abstract

**Introduction:**

Attention-deficit/hyperactivity disorder (ADHD) is a chronic neurodevelopmental condition that affects millions of children and adolescents worldwide. Knowledge of risk factors associated with ADHD may reduce its prevalence and its severe impact on patient’s quality of life. The aim of this study was to identify risk factors associated with ADHD and to discuss their involvement in the genesis of the disorder.

**Methods:**

This is a case-control study involving a first group of 74 children (mean age = 9 years) diagnosed with ADHD. The second group included 80 healthy control children. They were randomly selected and matched for age and gender. A literature-based questionnaire assessing the socio-demographic data, biological and environmental factors associated with ADHD was administered to the parents. The diagnosis of ADHD group was made by a trained child psychiatrist according to the DSM-5 criteria supplemented by the Conners scales of parents and teachers. For the control group, we added to the questionnaire the MINI-kid section of ADHD to screen for possible presence of ADHD symptoms. Univariate then multivariate analyses were conducted to identify factors associated with ADHD.

**Results:**

Several factors were more prevalent in children with ADHD than in controls: disturbed family dynamics, low socio-economic status, family history of psychiatric and organic pathologies, and particularly several early environmental factors, including passive smoking during pregnancy, prematurity, fetal distress, caesarean delivery and low birth weight. In the early childhood period, early exposure to television was also strongly associated with ADHD. However, the multivariate model conducted to determine the variables independently associated with ADD/ADHD revealed only three determining factors: passive smoking during pregnancy (OR = 4.60 [2.14, 9.94]; p < 0.001), acute fetal distress (OR = 5.08 [1.47, 17.52]; p = 0.01), and familial psychiatric history (OR = 9.37 [2.46, 35.59]; p = 0.001).

**Discussion:**

The recognition of factors involved in the genesis of ADHD within different ethnic populations may help understanding and broaden our knowledge of this disorder to develop targeted strategies for prevention and early intervention. Further participants with more robust statistical output are required to confirm our findings to a more generalized population.

## 1 Introduction

Attention-deficit/hyperactivity disorder (ADHD) is one of the most widespread psychiatric diseases worldwide, especially in children and adolescents (1). Psychiatric diseases often persist into adulthood, resulting in individual and collective social burden. Emerging evidence suggests that ADHD is among the biggest contributors to the global burden of disease in children and adolescents and evidence suggests that the cost of raising children with ADHD is considerably high when compared with raising children without such disorder (2, 3). Zhao et al. (4) found that the economic burden of raising children and adolescents with ADHD was five times higher than raising children without ADHD. Children with ADHD often have trouble with focus, lack self-control and exhibit impulsive or excessive behavior (5). Although this disorder has been studied for nearly a century and several hypotheses have been put forward, the cause and pathophysiology of ADHD is yet largely unknown. ADHD is a neurodevelopmental disorder that alter the children performance at school in terms of academic achievement and behavior (6–8). These impairments are the origin of sufferance and mismatch of the child and respectively his family. It is estimated that ADHD affects up to 8% of school-age children (6-12 years) worldwide, with a higher prevalence in boys (9, 10).

According to the recent review by Ayano et al. (11) with over 3 million participants, the global prevalence of ADHD in children and adolescents was 8.0%, 95% CI [6.0; 10]. The prevalence estimate was twice as high in boys (10%) compared to girls (5%). Of the three subtypes of ADHD, the inattentive type of ADHD (ADHD-I) was found to be the most common followed by the hyperactive (ADHD-HI) and the combined types (ADHD-C). A recent meta-analysis by Popit et al. (12) showed that the overall prevalence of ADHD in register studies was 1.6%, 95% CI [0.9; 3.0], in survey studies 5.0%, 95% CI [2.9; 8.6], in one-stage clinical studies 4.2%, 95% CI [2.9; 6.0], and in two-stage clinical studies 4.8%, 95% CI [4.0; 5.8]. The results from this systematic review and meta-analysis suggest that the type of study significantly affects the prevalence of ADHD.

In Tunisia, a previous estimated ADHD prevalence was 10% among school children in Sfax, a south-east region of Tunisia (13). This study was based on a screening using the Conners 2^nd^ version completed by a clinical psychiatric evaluation. A more recent study that screened adolescents in the region of Monastir, a central-east region in Tunisia revealed a prevalence of ADHD in 18% of the population (14). This second study has been limited to different aspects. First, there was selection bias due to the inclusion of only adolescents attending higher schools. Then, the Adult ADHD Self-Report Scale despite being translated into Arabic it was not validated in the Tunisian cultural context. The screening of ADHD in this study was solely based on the ASRS without additional clinical investigation.

In addition to this variability in ADHD prevalence worldwide, there is also a difference in drug therapy. There are many treatment challenges, including a low number of treated patients with stimulants and a low percentage of treated patients in some countries. ADHD is typically treated with pharmacotherapy and other interventions (e.g. cognitive behavioral therapy).

Stimulants are the first line treatment for ADHD in children, adolescents, and adults. Studies conducted in several countries have shown a significant increase in the use of ADHD drugs in the last years (15). These data are not available for many countries, for example some ex-Yugoslavian countries and Eastern Europe ones. In Slovenia, less than 50% of patients with ADHD were treated with medication. The number of patients treated with methylphenidate in the 6–12 age group remained approximately the same between 2007 and 2015. The use of atomoxetine is still higher and the percentage of diagnosed patients undergoing treatment is lower than in other developed countries (16). These data make the disorder even more complex.

ADHD is an ongoing and a serious expanding public health concern. In children, the disease is a major risk factor for substance misuse and the development of substance use disorder in adolescence and young adulthood, even when the most common childhood comorbidities such as conduct disorder and oppositional defiant disorder are controlled (17). As a result, multiple studies were initiated to determine its risk factors and to find an appropriate remedy. Previous studies uncovered evidence of genetic heterogeneity in the clinical presentations of ADHD (18) which is most probably a result of interaction of complex multilevel mechanisms (19).

Different etiological hypotheses of ADHD have been presented earlier, among these neurobiological and neurochemical theories are the most admitted. These studies confirmed dysregulation of the monoaminergic neurotransmission systems, including dopamine, serotonine and neropinephrine (20–22). In addition, a variety of neurological problems affecting the central nervous system (frontal lobe abnormalities, brain dysfunctions, and neurological immaturity) can cause behavioral difficulties associated with ADHD (23, 24). At the neuropsychological level, this disorder has been shown to be the result of a deficit in self-control and behavioral inhibition (25). The genetic predisposition of ADHD represents the most accepted hypothesis based on family, twins, and adoption studies (26, 27). It reinforces, therefore, the theory of biological predisposition.

Proposed ADHD environmental risk factors include prenatal substance exposure, nutritional factors and various complications during pregnancy (28–31). In addition, psychosocial factors including poverty, under-stimulation, physical or psychological abuse are also associated with greater symptoms of ADHD (32).

Taken together, some genetic and environmental factors seem to enhance ADHD predisposition which highly depend on the study population. Understanding the risk factors associated with genesis of ADHD may help in developing targeted strategies for the prevention of this disorder.

The aim of the present study was to screen for possible risk factors, particularly environmental and psychosocial related ones, associated with ADHD by comparing a cohort of age and sex-matched school-aged Tunisian population.

## 2 Materials and methods

### 2.1 Study design and data collection

This is a case-control study including a first group of children diagnosed with ADHD and a control group composed of children without ADHD, matched by age and gender.

### 2.2 Study area

This study was conducted in two University Hospitals from the central-east regions of Tunisia: 1) the University Hospital of Monastir, and 2) Tahar Sfar Hospital of Mahdia. The control group was recruited at the same period from two schools in the same region as the hospitals (Monastir and Mahdia).

### 2.3 Study population

We recruited over a period of 6 months spanning from October 2021 children with ADHD following at the Child and adolescent Psychiatry Out-patient Units. Criteria of inclusion were patients aged less or equal to 18 years old, having obtained the consent of the child’s parent to participate in this study. Criteria of exclusion were presence of autism spectrum disorder, moderate or severe intellectual disability or severe neurological disorder.

Inclusion criteria for controls were school children attending both schools at Monastir and Mahdia matched by age and sex to children with ADHD. In addition, the children must have obtained the consent of the child’s parent to participate in the study and the agreement of the school’s director. The exclusion criteria were the same as the ADHD group in addition to children whose have a positive screening of ADHD, evaluated by the ADHD section of Mini-International Neuropsychiatric Interview for Children and Adolescents (MINI-Kid). Controls were selected from the same region to minimize environmental variation. Two schools were selected using cluster sampling, first at the school and then at the class, followed by a random sample selection. The two groups were matched by age and gender.

The sample size was calculated using Biostat TGV, with β = 0.8, α = 0.05 and ratio of controls to cases = 1, OR of 3 and percentage of exposed controls of 20%. The calculated sample size was 73 children in each group.

### 2.4 Questionnaire

A pre-established questionnaire composed of six sections was administered to both parents of the groups. The items of the questionnaire were based on an intensive literature review and previously validated questionnaires within studies determining risk factors associated with ADHD of various ethnic populations [Poissant et al. (33), Tharpar et al. (6, 18), Banerjee et al. (29) and Taylor et al. (30)]. These risk factors were selected and included in the established Arabic version of the pre-established questionnaire (**Supplementary Data Sheet**). Before starting data collection, a preliminary version of the questionnaire developed was tested with around ten parents at the Child Psychiatry consultation in Monastir. Multiple modifications were thus made to this first version to conclude with a final version.

The first section contains 21 questions including pregnancy conditions (ages of the parents during pregnancy, the circumstances, and the progress of the pregnancy). The second section includes seven questions about the circumstances, progress of childbirth, and the health state of the newborn child. The third section is composed of six questions concerns the postnatal and early developmental periods [health condition of the mother and the infant, traumas, and premature exposure to television: less than the age of 2 years (34–36)]. The fourth section contains five questions evaluating the child’s developmental stages and his medical history. The fifth section (seven questions) tackles the early care of the child, his exposure to domestic violence and his school achievements. The last part of the questionnaire with 35 questions concerns sociofamilial characteristics, family pathological history and criminal records as well as the conjugal dynamics of the parents. For this questionnaire, we used closed-ended questions. The majority were binary types and the rest multiple-choice questions.

### 2.5 Diagnosis of ADHD

The 2^nd^ short version of Conners rating scales validated in Tunisian context were administered to children with ADHD, often during their first consultation, as a complement to the clinical examination to help to diagnose ADHD and to evaluate the severity of symptoms. Thus, the diagnosis of ADHD was made by an expert child and adolescent psychiatrist according to the DSM-5 criteria. For the control group, we added to the pre-established questionnaire, the MINI-kid section of ADHD to screen for possible presence of ADHD symptoms in “typical non-ADHD subjects”. This section contains 20 questions based on DSM-IV diagnostic criteria of ADHD. Using this section, we obtained 11 children with a positive screening of ADHD among the control group. These children were excluded from this study and referred for follow-up to the outpatient child and adolescent psychiatry unit.

The data of the control group were filled in by the parents in the school in the presence of the investigator or at home then returned to the director of the school.

### 2.6 Statistical analysis

Data analysis was conducted using SPSS (version 21.0 for Windows). Descriptive study was performed using effectives and percentages for qualitative variables, and mean and SD (standard deviation) for quantitative variables. For univariate analysis, the Chi-square test and Fisher’s exact test were used to probe for independent variables. The statistical significance was set at 5%.

A binary logistic regression was conducted to identify factors independently associated with ADHD in the multivariate model. Variables included in the multivariate analysis were those with a significance level below 25% in the univariate analysis.

## 3 Results

### 3.1 Sociodemographic characteristics

The ADHD population accounted for 74 children composed of 69% with a combined type, 12.3% with a predominant hyperactive-impulsive presentation, and 18.7% with an inattentive ADHD (**Table 1**). The age of ADHD children was 8.88 ± 2.26 (mean ± standard deviation [SD]) years with a sex ratio of 7.22 (male/female). Most ADHD children (78%) are under methylphenidate treatment and only 22% were without pharmacological supplementation. The control group was composed of 80 children with an age of 9.1 ± 2.1 (mean ± SD) and a sex ratio of 7. More than 91% of children (ADHD and controls) were at the primary school level and 9% were attending middle school. Most children with ADHD or without ADHD had an average socioeconomic level. The difference was not statistically significant (p = 0.055) for this variable between the two groups. Consanguinity between the parents was considered in 8% of the ADHD children and in 10.4% for the control group without a significant difference (p = 0.33).

**Table 1:** Demographic and clinical characteristics of ADHD group and non-ADHD controls.

	ADHD group	Control group
**Number**	74	80
**Age (mean ± SD)**	8.88 ± 2.26	9.08 ± 2.07
**Sex ratio (male/female)**	7.22	7
School level		
Primary school, n (%)	69 (93.2)	74 (92.5)
Middle school, n (%)	5 (6.8)	6 (7.5)
ADHD type		
Combined, n (%)	51 (69)	–
Inattentive, n (%)	14 (18.7)	–
hyperactive-impulsive, n (%)	9 (12.3)	–
Pathological history		
Organic pathology, n (%)	4 (5.5)	1 (1.3)
Psychiatric comorbidity*, n (%)	24 (32)	3 (3.7)

*Psychiatric comorbidity: elimination disorders, separation anxiety disorder, depression, specific learning disorders, and oppositional defiant disorder. SD, Standard Deviation; n, number; ADHD, Attention-deficit/hyperactivity disorder.

### 3.2 Pathological history

Psychiatric pathological familial history was associated with ADHD (p < 0.001, **Table 2**). Investigation of ADHD antecedents and other psychiatric disorders revealed that pathological history of anxiety, depression, and/or psychosis were more frequent in ADHD families (p = 0.005). For family history of ADHD, the difference between ADHD and control groups was significant in ADHD within the family members other than parents (p = 0.03) and in case of ADHD in parents (p = 0.01). A significant difference (p = 0.025) was also found for chronic organic family history. Children with ADHD had more psychiatric comorbidities than control children (p = 0.001), but no difference was reported between the two groups for organic comorbidities (p = 0.7).

**Table 2:** Association between familial characteristics and ADHD.

	ADHD n (%)	Control n (%)	p
Socioeconomic status			
Good Poor	52 (71.1) 22 (28.9)	67 (83.8) 13 (16.3)	0.055
Father’s educational level			
Illiterate and primary school High school and university	16 (21.6) 58 (78.3)	20 (25) 60 (75)	0.19
Mother’s educational level			
Illiterate and primary school High school and university	20 (27) 54 (72.9)	10 (12.5) 70 (87.6)	0.009
**Physical and emotional violence**	22 (29.7)	26 (32.6)	0.18
**Disrupted family dynamics**	21(28.4)	16 (20)	0.11
Mother’s age during pregnancy			
≤25 >25	19 (25.7) 55 (74.3)	21 (26.3) 59 (73.8)	0.54
Father’s age during pregnancy			
≤35 >35	39 (52.7) 48 (60)	35 (47.3) 32 (40)	0.36
**Consanguinity**	6 (8.1)	5 (10.4)	0.33
**Familial psychiatric history**	25 (33.8)	7 (8.8)	<0.001
**ADHD in other members of the family**	3 (4.1)	0 (0)	0.03
**ADHD in parents**	5 (6.8)	0 (0)	0.01
**Chronic organic family history**	28 (37.8)	19 (23.8)	0.025

p, p-value; n, number; ADHD, Attention-deficit/hyperactivity disorder.

### 3.3 Psycho-social characteristics

Family problems including parental separation, divorce, conflicts, and financial problems were higher in children with ADHD. However, the differences were not statistically significant for these variables: intrafamilial verbal and physical violence, and family problems, with p = 0.18 and p = 0.11, respectively. For the parents’ educational level, ADHD was associated with a lower level (illiterate and primary) in mothers (p = 0.009), but not in fathers (p = 0.19). The age of parents during the pregnancy did not correlate with ADHD (p = 0.54 for mothers and p = 0.36 for fathers). There were no criminal records reported by the parents.

### 3.4 Circumstances of pregnancy and delivery

The univariate study showed an association between ADHD and exposition to passive smoking (p < 0.0001), cesarean delivery (p = 0.02), low birth weight (p = 0.05), preterm birth (p = 0.01) and acute fetal distress (p = 0.002, **Table 3**). The other studied variables related to perinatal factors were not associated with ADHD. Neither active smoking (p = 0.14), alcohol consumption (p = 0.71) nor use of other psychoactive substances during pregnancy associated with ADHD. None of mothers reported consuming any other type of psychoactive substances during pregnancy. No association was found between ADHD and twin pregnancy (p = 0.32) nor induced pregnancy by an *in vitro* fertilization (p = 0.48). Exaggerated sympathetic signs during pregnancy such as unusual fatigue, nausea, constipation, heartburn, sensitivity to odors and mood changes were equally present between both groups without significantly different results (p = 0.35). Similarly, the unusual prolongation of sympathetic signs beyond the first trimester of pregnancy did not correlate with ADHD (p = 0.5). There was no association between ADHD and the presence of genital bleeding during pregnancy (p = 0.09) with all causes combined: bleeding from implantation, hormonal dysregulation, genital infection, or sexual intercourse. Concerning the threat of abortion or premature delivery, this complication was reported with close proportions between the two groups (p = 0.36). Similarly, neither the presence of urogenital infections during pregnancy (low urinary tract infection, acute pyelonephritis, urethritis, cervicitis, p = 0.41) nor gestational diabetes (p = 0.27) were associated with ADHD. In the case of pregnancy toxemia, the control group had the highest percentage, but the difference remained insignificant (p = 0.35) between ADHD and control groups. Concerning medication during the pregnancy, there was also no association between ADHD and the intake of any medications, including analgesic, anti-inflammatory, anti-hypertensive and anti-diabetic (p = 0.26).

**Table 3:** Association between perinatal and postnatal characteristics and ADHD.

	ADHD group n (%)	Control group n (%)	p
***In vitro* fertilization**	1 (1.4)	1 (1.3)	0.48
**Twin pregnancy**	5 (6.8)	4 (5)	0.32
**Exaggerated sympathetic signs**	20 (27)	20 (25)	0.35
**Prolonged sympathetic signs**	5 (6.8)	12 (15)	0.5
**Gestational diabetes**	38 (51.4)	16 (20)	0.27
**Urogenital infections**	4 (5.4)	5 (6.3)	0.41
**Toxemia**	2 (2.7)	3 (3.2)	0.35
**Genital bleeding**	13 (17.6)	8 (10)	0.09
**Threat of abortion or premature delivery**	10 (13.5)	9 (11.3)	0.36
**Passive smoking**	36 (48.6)	16 (20)	< 0.001
**Active smoking**	4 (6)	1 (1.3)	0.14
**Alcohol consumption**	1 (1.5)	1 (1.3)	0.71
**Medication during pregnancy**	14 (18.9)	12 (15)	0.26
**Cesarean delivery**	20 (27)	11 (13.8)	0.02
**Prematurity**	7 (9.5)	1 (1.3)	0.01
**Acute fetal distress**	15 (20.3)	4 (5)	0.002
**Low birth weight**	9 (12.2)	4 (5)	0.05
Season of birth			
Cold season	41 (55.4)	34 (42.5)	0.11
Hot season	33 (44.6)	79 (51.3)	
**Postpartum depression**	8 (11.9)	4 (5.2)	0.12
**Head injury < 2 years**	8 (10.8)	2 (4.2)	0.09
**Early exposition to TV < 2 years**	28 (37.8)	19 (23.8)	0.025

p, p-value; n, number; ADHD, Attention-deficit/hyperactivity disorder.

### 3.5 Postnatal and early developmental period

The mothers with ADHD had more postpartum depression after childbirth than controls, however, the relationship between ADHD and the presence of depression was not statistically significant (p = 0.12, **Table 3**). The same result was found for cranial trauma during the first two years of life (p = 0.09). All the reported traumas were mild and without immediate complications. During their first two years of life, children with ADHD were more exposed to TV than children without ADHD and the difference between both groups was significant (p = 0.025).

### 3.6 Logistic regression analysis

A multivariate regression model was used to determine variables independently associated with ADHD. Three factors were found to be associated with ADHD: passive smoking during pregnancy, acute fetal distress and familial history of psychiatric disorders (see **Table 4**).

**Table 4:** Results of multivariate analysis on independently associated factors to ADHD.

	OR	CI	p
Passive smoking during pregnancy	4.60	[2.1, 9.9]	<0.001
Acute fetal distress	5.08	CI [1.4, 17.5]	0.01
Familial history of psychiatric disorders	9.37	[2.4, 35.5];	<0.001

OR, Odds ratio; CI, Confidence interval; p, p-value.

## 4 Discussion

Neurodevelopmental disorders, particularly ADHD, have become in recent years increasingly a subject of interest as to their etiologies. It remains, however, a complex task in which investigators must face various methodological issues. An additional challenge is that some studies tend to dissociate factors of ADHD into two broad areas: genetic and environmental/social (37). Nevertheless, it seems that ADHD highly depends on these both components which continuously influence each other. This study aimed to identify risk factors associated with ADHD in a Tunisian population with a focus on environmental and psychosocial related ones by comparing them to a group of healthy controls.

Passive smoking, prematurity, history of acute distress, and cesarean delivery were positively correlated with ADHD in the univariate analysis. A French mother-child cohort study found that the risk of developing ADHD symptoms at age of five increases with daily cigarette consumption during pregnancy (38). While in a Chinese child and adolescent cohort, parental smoking before and throughout pregnancy was a risk factor for offspring developing ADHD (39). In the current study, we did not find an association between ADHD and active mother’s smoking. This can be explained by the insubstantial number of mothers reporting smoking, which may be due to methodological limitations. For passive maternal smoking, Langley et al. (40) showed that symptoms of hyperactivity-impulsivity were significantly higher in ADHD children of exposed mothers, which is in line with our results. Some hypotheses have been put forward for the mechanism by which tobacco acts on the risk of ADHD development. The toxins contained in tobacco, especially nicotine, can pass directly through the placenta and act on the child nervous system (41), through triggering of dopamine release (42). Similarly, tobacco smoke can induce an increase in umbilical blood flow resistance and subsequently cause fetal distress (43). Regardless of the mechanism, the impact of smoking would depend on a genetic fetus’s predisposition (44). Alcohol consumption during pregnancy has also been linked to ADHD in multiple studies (45, 46). This factor was not investigated in this study due to the low number of mothers reporting alcoholism. In line with the previous study by Perapoch et al. (47) and the meta-analysis conducted by Franz et al. (48), our result revealed that low birth weight and prematurity were positively correlated with ADHD in the univariate analysis. In this context, Chu et al. (49) found that moderate prematurity and low birth body weight were associated with ADHD in a Taiwanese population. Both parameters act by a multifactorial mechanism including intrauterine growth retardation and a less mature brain structure (50). One of the possible intermediates between ADHD, preterm and low birth weight is also prenatal anoxia involved in the genesis of ADHD (51). Anoxia is the cause of acute fetal distress, which was found in our study as an independent risk factor of ADHD (52). It can be secondary to many perinatal conditions such as obstetric complications. Thus, in the current study, cesarean delivery was positively correlated with ADHD at the univariate analysis. Several hypotheses have been put forward to explain this possible association, including exposure to a modified microbiota (53–55), and inadequate priming of the stress response (56).

With regard to postnatal factors, we did not detect a correlation between blunt head trauma and ADHD in infants, while others suggested a possible relationship (57). Reciprocally, early exposure to television (before age of 2 years) was associated with ADHD in the univariate study. Attention disorders appear to correlate with early screen time (58). Similarly, daily exposure to television before age of two is associated with symptoms of hyperactivity-inattention at young age (59). Symptoms of hyperactivity are often higher in children with impaired mother-baby interactions and less family support (60). Maternal depression was shown to be among the predictive factors of the persistence of hyperactivity symptoms in children (61). In this study, mothers of children with ADHD reported more postpartum depression without a statistically significant association, probably because of information bias.

For psychosocial risk factors, we did not identify a relationship between ADHD and low socioeconomic status, exposure to parental violence and disrupted family dynamics. The socioeconomic level is one of the indicators of psychosocial adversity documented in multiple studies. A high socioeconomic status would usually provide a healthy and enriched environment for better familial management of difficult behaviors (62). Parent’s education levels and attitude toward ADHD symptoms remains a less studied subject with inconclusive results (63). In the current study, fathers’ educational status was not associated with ADHD, but a higher level of mothers was inversely associated with ADHD. A possible explanation is that mothers are often the ones bearing the greatest burden in managing their children within the Tunisian context. These parenting practices can also be influenced by the age of the parents. Previous investigations on this subject are inconclusive, even though some studies revealed that young mother’s age is related to ADHD (64). Recently, in a meta-analysis of 12 studies published in December 2024, Zhao et al. (65) identified a significant association between preterm birth and maternal education levels with the risk of developing ADHD in children. Subgroup analysis further suggested that this protective effect of maternal education was particularly significant in studies conducted in China (OR = 0.59, 95%CI: 0.46-0.75, P < 0.001, I² = 81.2%), while no significant association was observed in studies from other regions (OR = 1.25, 95%CI: 0.66-2.40, P = 0.495, I² = 92.3%). This comprehensive evaluation highlights the importance of both biological and socioeconomic factors in the development of ADHD in children. The findings from this meta-analysis underscore the importance of risk mitigation strategies for premature infants and children of mothers with low educational attainment. Healthcare providers should prioritize early identification and support for these high-risk groups.

In this study, there was no direct correlation between physical or emotional intrafamilial violence and ADHD. Based on previous investigations, disturbed family dynamics is an indication for symptoms severity in children with ADHD (66). Thus, persistence of ADHD is predictable from psychosocial adversity and psychiatric comorbidity (67).

Family psychiatric history was among the independent risk factors of ADHD found in the current study. There is convincing evidence of a strong hereditary contribution to ADHD. Family studies consistently revealed higher incidence of ADHD among parents and siblings of ADHD subjects compared to parents of unaffected controls (68). Twin studies have shown that monozygotic twins have much higher concordance rates for ADHD than dizygotic ones (69). Adoption studies have also confirmed increasing percentages of ADHD in the biological parents of adopted children with ADHD (70, 71). Lichtenstein et al. (72) estimated genetic effects at 79% for ADHD suggesting that nonhereditary factors can also contribute. ADHD also shares hereditary responsibility with other neurodevelopmental and psychiatric problems including autism spectrum disorders and developmental coordination disorders. This may partly explain the high psychiatric comorbidity in children with ADHD (73–75), which was objectified in our study.

The results of the current study revealed a significant trigger of perinatal, psychosocial, and genetic factors in the etiopathogenesis of ADHD. A complex interaction between environmental and genetic factors has been demonstrated earlier (26, 27, 76, 77). Brain developmental theory suggests that environmental conditions interact with biological risk factors throughout child development stages (78). Gene expression and brain plasticity may be more sensitive to environmental inputs during development (79). Therefore, early childhood adversity may have more lasting effects on the brain. Genetic risks may not only contribute directly, but they may also work by increasing the likelihood of exposure to environmental adversity and changing the sensitivity to environmental risks and protective factors. Environmental factors can also influence early and later development in a more dynamic way by influencing the gene expression which is known as epigenetics. This type of complex interaction in early life may amplify or modify the risk of ADHD (80–82).

The findings of the current study were important given the growing prevalence of ADHD in our country, and north Africa in general. As part of the search for etiopathogenic factors, these results will lead to a better understanding of the disorder and importantly to the implementation of prevention strategies and the development of new research in the field. This study may offer possibilities for intervention and perhaps prevention to improve the environment and reduce the risk factors for ADHD by controlling certain factors such as acute stress and passive smoking during pregnancy. These results will raise awareness of the need to screen at-risk pregnancies in order to ensure better medical follow-up and provide the necessary perinatal support.

This study has some methodological limitations. First, the retrospective collection of data is a source of memory bias, second, using a self-questionnaire can cause information bias. The small size of our sample reduces the strength of the statistical results, hence the interest in continuing with prospective longitudinal studies with a larger population. Despite this, our results confirm the multiplicity and complexity of factors associated with ADHD. The second step of our study will continue a large-scale prospective study in multiple hospitals. In addition, genetic and epigenetic studies are also required to better characterize the vulnerability of ADHD in children. Such studies can offer opportunities for intervention and maybe prevention to improve the environment and reduce risks of ADHD by controlling some factors such as maternal stress during pregnancy, smoking practices and negative interactions with parents.

## 5 Conclusion

Several risk factors including passive smoking, prematurity, history of acute distress, and cesarean delivery were positively correlated with ADHD of a Tunisian population. Case-control study seems to be a good option for evaluation since ADHD is present only in a minor fraction of the population. Our objective was to determine risk factors that have an association with this neurodevelopment disorder.

## Data Availability

The raw data supporting the conclusions of this article will be made available by the authors, without undue reservation.
