# Supplementary material for: Factors associated with attention-deficit/hyperactivity disorder among Tunisian children
Source: Front Psychiatry. 2025 Feb 7;16:1462099. doi: 10.3389/fpsyt.2025.1462099 (PMC11842382; doi:10.3389/fpsyt.2025.1462099)
Supplement: Supplementary file 1 [file DataSheet1.pdf]

frontiers

Supplementary Material

Ministry of Public Health
Monastir University Hospital
Child and Adolescent Psychiatry Department
Tel: 73461144 / 73106000

This questionnaire is part of the preparation for a doctoral thesis in medicine on Attention Deficit Hyperactivity Disorder (ADHD) in school-aged children. All researchers involved in this study commit to ensuring the complete confidentiality of the information provided in the questionnaire

Data Collection Sheet
Attention Deficit Hyperactivity Disorder (ADHD) and Environmental Factors in Children

1- Name and surname: .... *(optional)*
2- Age: ....
3- Gender: Male ☐ Female ☐
4- Educational level: ....
5- Phone number: .... *(optional)*

Pregnancy Period

1- Mother's age at the time of pregnancy with the child:
....

2- Father's age at the time of pregnancy with the child:
....

3- Natural pregnancy: Yes ☐ No ☐ If yes, in which month did it occur?
....

4- Twin pregnancy: Yes ☐ No ☐ If yes, in which month did it occur?
....

5- IVF (In vitro fertilization): Yes ☐ No ☐ If yes, in which month did it occur? ....

6- Use of medication to achieve pregnancy: Yes ☐ No ☐ If yes, in which month did it occur? ....

7- Presence of more than usual pregnancy symptoms (e.g., vomiting): Yes ☐
No ☐
If yes, how long did they last? ....

8- Presence of illness during pregnancy:

BEN SALEM Nizar • Tel: 50 [illegible] 680 • Monastir • Sworn Translator

- Gestational diabetes: Yes ☐ No ☐
- Bleeding during pregnancy: Yes ☐ No ☐
- Infection during pregnancy: Yes ☐ No ☐
- High blood pressure/preeclampsia during pregnancy: Yes ☐ No ☐
- Other illnesses: Yes ☐ No ☐ *If yes, specify:* ....

9- Possibility of preterm birth: Yes ☐ No ☐
10- Nausea and pregnancy symptoms after the first trimester: Yes ☐ No ☐

11- Weight gain of more than 25 kg: Yes ☐ No ☐
12- Weight gain of more than 10 kg: Yes ☐ No ☐
13- Any accident involving the mother: Yes ☐ No ☐
14- Use of medications during pregnancy: Yes ☐ No ☐ *If yes, which ones?*
....
15- Alcohol consumption during pregnancy: Yes ☐ No *If yes, amount per day:* ....
16- Tobacco use during pregnancy: Yes ☐ No ☐ *If yes, how many cigarettes per day?* ....
17- Passive smoking (a family member smoking near you): Yes ☐ No ☐
18- Drug use during pregnancy: Yes ☐ No ☐ *If yes, what and how much per day?* ....

19- In general, did the mother require medication or hospitalization during pregnancy? Yes ☐ No ☐
If yes, specify the reason:
....

20- Presence of violence, marital conflicts, or other family problems during pregnancy: Yes ☐ No ☐
21- Separation or divorce of the parents: Yes ☐ No ☐

Birth

1- Was the birth:

- Natural birth Yes ☐ No ☐
- Cesarean birth Yes ☐ No ☐ *If yes, what was the reason?* ....
- Use of medications (synto/cytotec) Yes ☐ No ☐
- Use of instruments (forceps) Yes ☐ No ☐

2- Complications during birth: Yes ☐ No ☐ *If yes, what were they?* ....
3- Premature birth: Yes ☐ No ☐ *If yes, what was the reason?*
....
At what month? ....

BEN SALEM Nizar · Tél: [illegible] 881 680 · Monastir · Sworn Translator

4- Season in which the birth occurred:
Summer ☐ Autumn ☐ Winter ☐ Spring ☐
5- Weight at birth: ....
6- Did the baby experience distress at birth?

- No ☐
- Did not cry at birth ☐
- Born blue in color ☐ ☐
- Other ....

7- Did the baby require hospitalization? Yes ☐ No ☐

- *If yes, why?* ....
- *How long was the stay?*
  ....
- *Did the mother breastfeed the baby during this period?*
  ....

Post-birth

1- Meningitis/encephalitis in the child: Yes ☐ No ☐

- *If yes, at what age?* ....

2- Other complications in the child: Yes ☐ No ☐

- *If yes, what were they?* ....

3- Was the child admitted to the hospital? Yes ☐ No ☐

- *If yes, at what age?* ....
- *Why?* ....

4- Head trauma (fall/accident): Yes ☐ No ☐

- *If yes, at what age?* ....

5- Did the mother experience postpartum depression? Yes ☐ No ☐

6- Did the child watch TV before the age of two? Yes ☐ No ☐

- *If yes, for how long?* ....

Child Development and Health Status

1- Did you notice that your child's growth was normal and then delayed or stopped at a certain age?
Yes ☐ No ☐ *If yes, at what age?* ....

BEN SALEM Nizar · Tél: 98 381 680 · Monastir · Sworn Translator

2- How was the child's sleep:

- Normal ☐
- Too calm ☐
- Restless ☐
- Fearful ☐
- Other sleep disorders, what were they? ....

3- Does the child suffer from:

- Physical illnesses Yes ☐ No ☐ *If yes, what are they?* ....
  ....

- Learning difficulties Yes ☐ No ☐
- Depression Yes ☐ No ☐
- Intellectual disability Yes ☐ No ☐
- Suicide attempt Yes ☐ No ☐
- Epilepsy Yes ☐ No ☐
- Sleep disorders Yes ☐ No ☐
- Urinary incontinence (light/heavy) Yes ☐ No ☐

4- Does the child take any medications? Yes ☐ No ☐ *If yes, what are they?* ....

5- If there are any other psychological issues, please mention them: ....

Educational and School Environment*

1- Who primarily took care of the child's upbringing? ....

2- Was there anyone else who took care of the child besides the parents? (Nanny, housemaid, grandmother, etc.)

....

3- When was the child enrolled in:

- Nursery: ....
- Quran school: ....
- Other: ....

4- How many hours did the child spend in nursery/school? ....

5- How many hours does the child currently spend on:

- TV? ....
- Computer? ....
- Tablet (I-Pad)? ....
- Video games (PlayStation)? ....

BEN SALEM Nizar · Tél: 381 680 · Monastir · Sworn Translator

- Internet? ....

6- Has the child been exposed to family violence:

- Verbal ☐ Physical ☐

7- Child's academic performance:

Overall Average	Writing		Reading		Written Expression		Oral Expression		Mathematics
	Arabic	French	Arabic	French	Arabic	French	Arabic	French	

Family History

1- Social status: Poor ☐ Average ☐ Good ☐ Very good ☐

2- Who does the child live with in the household?

....

Mother

1- Age ....

2- Educational level ....

3- Occupation ....

4- Mental illnesses: Yes ☐ No ☐ *If yes, what are they?*

....

5- Physical illnesses: Yes ☐ No ☐ *If yes, what are they?*

....

6- Takes medication: Yes ☐ No ☐ *If yes, what are they?*

....

7- Consumes alcohol: Yes ☐ No ☐ *If yes, what is the amount per day?*

....

8- Consumes tobacco: Yes ☐ No ☐ *If yes, how many cigarettes per day?*

....

9- Uses drugs: Yes ☐ No ☐ *If yes, what is the amount per day?*

....

10- Has a criminal record: Yes ☐ No ☐

Father

1- Age ....

2- Educational level ....

3- Occupation ....

4- Mental illnesses: Yes ☐ No ☐ *If yes, what are they?*

SALEM Nizar Tél: ... 381 680 Monastir Sworn Translator

....
5- Physical illnesses: Yes ☐ No ☐ *If yes, what are they?*
....
6- Takes medication: Yes ☐ No ☐ *If yes, what are they?*
....
7- Consumes alcohol: Yes ☐ No ☐ *If yes, what is the amount per day?*
....
8- Consumes tobacco: Yes ☐ No ☐ *If yes, how many cigarettes per day?*
....
9- Uses drugs: Yes ☐ No ☐ *If yes, what is the amount per day?*
....
10- Has a criminal record: Yes ☐ No ☐

The Couple

1- Is there any relation between the parents? Yes ☐ No ☐ *If yes, what is it?*
....
2- Violence between the couple: Verbal ☐ Physical ☐
3- Is there a separation between the couple? Yes ☐ No ☐ *If yes, how old was the child at that time?* ....
4- Is there a divorce? Yes ☐ No ☐ *If yes, how old was the child at that time?* ....
5- Emigration outside Tunisia? Yes ☐ No ☐ *If yes, how old was the child at that time?* ....

Siblings

1- Number of siblings ....
2- Child's order among siblings ....
3- Does any sibling suffer from a physical illness? Yes ☐ No ☐ *If yes, what is it?* ....
4- Does any sibling suffer from a mental illness? Yes ☐ No ☐ *If yes, what is it?* ....
5- Does any sibling have learning difficulties? Yes ☐ No ☐

Extended Family

1- Does any member of the extended family suffer from a physical illness? (e.g., uncles, aunts, grandparents...) Yes ☐ No ☐ *If yes, what is it?*
....
2- Does any member of the extended family suffer from a mental illness? Yes ☐ No ☐ *If yes, what is it?* ....
3- Does any member of the extended family have a criminal record? (e.g., imprisonment) ....

BEN SALEM Nizar · Tél: · Monastir · Sworn Translator
